# Spinal degeneration is associated with lumbar multifidus morphology in secondary care patients with low back or leg pain

**DOI:** 10.1038/s41598-022-18984-1

**Published:** 2022-08-29

**Authors:** Jeffrey R. Cooley, Tue S. Jensen, Per Kjaer, Angela Jacques, Jean Theroux, Jeffrey J. Hebert

**Affiliations:** 1 College of Science, Health, Engineering and Education, Murdoch University, 90 South Street, Murdoch, WA 6150 Australia; 2 Department of Diagnostic Imaging, Regional Hospital Silkeborg, Silkeborg, Denmark; 3 Chiropractic Knowledge Hub, Odense M, Denmark; 4 Spine Centre of Southern Denmark, Middelfart, Denmark; 5 Department of Sports Science and Clinical Biomechanics, University of Southern Denmark, Odense M, Denmark; 6 Health Sciences Research Centre, UCL University College, Odense M, Denmark; 7 Institute for Health Research, University of Notre Dame Australia, Fremantle, WA Australia; 8 Faculty of Kinesiology, University of New Brunswick, Fredericton, NB Canada

**Keywords:** Epidemiology, Musculoskeletal system, Magnetic resonance imaging

## Abstract

Associations between multifidus muscle morphology and degenerative pathologies have been implied in patients with non-specific low back pain, but it is unknown how these are influenced by pathology severity, number, or distribution. MRI measures of pure multifidus muscle cross-sectional area (CSA) were acquired from 522 patients presenting with low back and/or leg symptoms in an outpatient clinic. We explored cross-sectional associations between the presence, distribution, and/or severity of lumbar degenerative pathologies (individually and in aggregate) and muscle outcomes in multivariable analyses (beta coefficients [95% CI]). We identified associations between lower pure multifidus muscle CSA and disc degeneration (at two or more levels): − 4.51 [− 6.72; − 2.3], Modic 2 changes: − 4.06 [− 6.09; − 2.04], endplate defects: − 2.74 [− 4.58; − 0.91], facet arthrosis: − 4.02 [− 6.26; − 1.78], disc herniations: − 3.66 [− 5.8; − 1.52], and when > 5 pathologies were present: − 6.77 [− 9.76; − 3.77], with the last supporting a potential dose–response relationship between number of spinal pathologies and multifidus morphology. Our findings could hypothetically indicate that these spinal and muscle findings: (1) are part of the same degenerative process, (2) result from prior injury or other common antecedent events, or (3) have a directional relationship. Future longitudinal studies are needed to further examine the complex nature of these relationships.

## Introduction

Low back pain (LBP) is a ubiquitous clinical complaint, usually non-specific in nature, with an estimated cost in the billions recorded by numerous countries^1^. It ranks in the top three causes of years lived with disability in every global region^2^. While there have been extensive research efforts attempting to enhance our understanding of the causes and most effective treatments for non-specific LBP, a full understanding of this disorder continues to prove elusive. Initial attempts to understand LBP often used pathoanatomic, pathomechanical, and/or biological approaches to identify discrete pain generating tissues, but by the end of the twentieth century this approach appeared to have failed in providing a clear connection^3–6^. Still lacking a clear alternative model, a renewed interest in investigating the biologic determinants of LBP has occurred^7^.

As a result, since the turn of the century there has been an ever-increasing interest in assessing the paraspinal muscles (and in particular the lumbar multifidus muscle (LMM)) to determine the role they might play in the cause, management and outcomes of patients with non-specific low back and/or leg pain^8^. But for this to be fully addressed, any potential underlying associations between neuro/pathoanatomic changes and altered LMM morphology (e.g., atrophy, fat replacement) require further exploration. For example, biopsy evidence suggests that ongoing neurocompressive effects from a disc herniation on the nerve root could result in alternations to the LMM fibres supplied by that nerve^9^, and imaging evidence suggesting herniations in patients with chronic radiculopathy can be associated with increased fatty replacement of the LMM^10^. However, the evidence in this area is still inconclusive.

Other issues also cloud our understanding of the associations between MRI-identified lumbar region degenerative findings, LMM changes, and clinical presentations. While it is generally acknowledged that degenerative MRI findings will be found in both symptomatic and asymptomatic individuals across all age ranges, the prevalence of these changes does appear to increase with age^11^. Additionally, a relationship between lumbar degenerative disorders and LBP, beyond what can be explained by the high prevalence of these disorders, has been noted. This relationship was particularly noted as disease severity increased, but it was also present in younger populations^11,12^. How some of these degenerative processes (e.g., those affecting spinal joints, vertebrae, or alignment) may relate to alterations in lumbar paraspinal muscle morphology has been investigated in numerous studies^10,13–25^, but these have typically focussed on assessing specific types or focused combinations of pathology, provided generic pathology comparisons, or utilized smaller population samples. None have looked at the aggregate effect of numerous pathologies.

Even where associations between altered LMM morphology and the presence of specific lumbar region pathologies have been implied, additional questions remain: Are these associations most strongly noted with each pathology in isolation; will the severity or combination of different MRI-identified pathologies also impact these associations; could specific combinations of altered muscle morphology and regional pathology help select the most cost-effective treatments? If we can identify, or begin to rule out, any associations between degenerative pathologies and LMM morphology, this may help us better understand how, or if, these work in concert to contribute to any associations with low back or leg pain. Therefore, the primary objective of this study was to explore the cross-sectional associations of discovertebral and facet joint-related degenerative lumbar MRI findings, both individually and in combination, to LMM morphology in patients presenting with a lumbar-related complaint. We hypothesized that bivariate associations will be demonstrated between certain types and/or combinations of lumbar region pathology and lower proportions of pure multifidus muscle. A secondary objective was to assess the intra-examiner reliability of a revised LMM measurement method.

## Material and methods

Overall approval for this project was granted by the Murdoch University Human Research Ethics Committee (approval: 2017/110). Components of this analysis (imaging; pathology coding) utilized existing data collected for a cohort study approved by the Danish Data Protection Agency for the Region of Southern Denmark (Journal number: 2008-58-0035-15/22513), acquired following the Declaration of Helsinki principles, including informed consent. Standard EU contractual clauses and data processor agreements between Murdoch University and the Region of Southern Denmark (18/22277) were established to allow for access of patient data.

### Study sample

Patients who presented to the Spine Centre of Southern Denmark, Hospital Lillebaelt, between September 2013 to October 2014, with a primary complaint of low back and/or lower extremity symptoms were recorded in the SpineData registry^26^. Those patients who also completed questionnaires for low back and leg pain at their initial visit and who had acquired lumbar MRIs within 30 days of this visit from the Hospital's radiology department were initially included for evaluation, and those with MR images pre-coded for spinal pathology met the final inclusion criteria for pathology/muscle analysis.

### MRI protocols

All patients underwent a standardized MRI protocol on one of two systems using a body/spine coil: 1.0 T Philips Panorama (Best, The Netherlands) or 1.5 T Philips Achieva (Best, The Netherlands). Sagittal T1-weighted turbo spin echo (TSE) and axial T2-weighted TSE (angled along the L3/4–L5/S1 disc planes) sequences were used. As T2-weighted sequences have been shown to be equivalent to T1-weighted sequences for the purposes of evaluating LMM cross-sectional area (CSA)^8^, T2 axial images were used for these measures, with the sagittal views used to assist with level localization.

### MRI pathology selection, coding, and assessment criteria

Consecutive MRIs had been previously selected from the earliest group of patients meeting the initial inclusion criteria, then assessed and coded for the presence and characteristics of any spinal pathology. Each assessment was performed individually by one of three trained clinicians with 5 + years of clinical spinal MRI experience. An experienced senior musculoskeletal research radiologist provided additional training in the identification and grading of various MRI findings using an evaluation manual, followed by consensus meetings. During training, an inter-rater reliability study (n = 50) assessed between the three raters and the radiologist on several conditions, noting moderate to strong reliability for disc degeneration (DD), high-intensity zones (HIZ), disc herniation, Modic type 1, and facet degeneration (i.e., arthrosis) (Kappa values ranging from 0.60 to 0.81). However, only minimal to weak reliability was noted for nerve root compromise, Modic type 2, and disc bulge (Kappa values from 0.29 to 0.58).

From this dataset, the following degeneration-related conditions were used for our study: DD, disc bulge, disc herniation (including any associated nerve root compromise), HIZ, and endplate defects (i.e., Schmorl’s nodes), as classified by Fardon et al.^27^; Modic marrow changes, as classified by Jensen et al.^28^; vertebral body osteophytes and facet arthrosis. Specific assessment criteria for this study were developed for individual and combined pathology analyses (Table 1). An approach adapted from Hancock et al.^29^, was used to combine MRI-identified pathologies for aggregate analysis, in order to investigate the effect of multiple degenerative findings being present concurrently per case. Pathologies were initially recorded from T12-S1 for most variables; however, as few positive findings were present at T12/L1 or L1/L2, these levels were not included in the final analyses.

**Table 1:** Pathology variables and assessment criteria.

Pathology type	Assessment criteria (individual)^1^
Disc degeneration	Condensed Pfirrmann grading (0 = none (0); 1 = slight (1); 2 = moderate (2) to severe (3))
Disc bulge	Present or absent at any level (0 = absent; 1 = present)
Disc herniation	Herniation grading based on any associated NR compromise (grade 0 = no herniation/herniation with no NR compromise (0) or NR contact only (1); grade 1 = displaced NR (2) or compressed NR (3)); herniation location based on apex location: right-sided, central, or left-sided
High intensity zone (HIZ)	Number of levels with HIZ present (0 = none; 1 = one level; 2 = two or more levels)
Modic marrow changes	Modic 1 and 2 changes (upper or lower) assessed separately; graded by maximum height of marrow change (0 = none (0) to < 25% (1); 1 = ≥ 25% (2) to ≥ 50% (3))
Endplate defects	Present or absent at any level (either endplate) (0 = absent; 1 = present)
Vertebral osteophytes	Number of levels with osteophyte(s) present (0 = none; 1 = one level; 2 = two or more levels)
Facet arthrosis (L3/4–L5/S1 only)	Number of levels with grade 1 arthrosis present^2^ (0 = none; 1 = one level; 2 = two or more levels)

Pathology type	Assessment criteria (combined)^3^
Disc degeneration	Worst condensed Pfirrmann grading (0–3) at any level (0 = 0–1 grading; 1 = 2–3 grading)
Disc bulge	Present or absent at any level (0 = absent; 1 = present)
Disc herniation	Worst sub-grading (0–3) at any level or location (0 = 0–1 sub-grading; 1 = 2–3 sub-grading)
High intensity zone	Present or absent at any level (0 = absent; 1 = present)
Modic marrow changes	Worst Modic 1 or 2 sub-grading (0–3) at any level (0 = 0–1 sub-grading; 1 = 2–3 sub-grading)
Endplate defects	Present or absent at any level (0 = absent; 1 = present)
Vertebral osteophytes	Present or absent at any level (0 = absent; 1 = present)
Facet arthrosis	Worst arthrosis grading (0–1) at any level (0 = 0–1 finding; 1 = 2 + findings)

^1^Each pathology assessed from L2/3 through L5/S1 unless otherwise indicated. ^2^Grading based on number of different degenerative findings (out of 8) present (grade 1 = two or more findings). ^3^Each pathology scaled at 0 or 1 to standardize analysis of any combinations. NR, nerve root.

### MRI muscle measurements

Axial measures were taken from a single slice located at the level of the disc or along/near the lower endplate (lower 1/3rd of the foramen), depending on which provided the fullest and clearest posterior arch anatomy and LMM outlines. All measures were acquired at L4/5 and L5/S1 bilaterally. Patients were excluded if their images showed obvious or apparent surgery from L4-S1, spinal cancer or acute fracture, insufficient image quality, only partial visualization of the LMM at either level, slice overlap artefact through the LMM, distorted or unidentifiable posterior arch anatomy, or did not include the L4/5 or L5/S1 level or T2-weighted axial images.

All LMM measurements were performed utilizing sliceOmatic v5.0.8b [TomoVision, Magog, Canada], as this system allows for outlining muscle CSA with specific quantification of the contained tissues, as well as histogram analysis functionality. All measurements were undertaken by a clinical researcher with over 30 years of experience in spinal MRI interpretation, previous experience using sliceOmatic software in LMM analysis, and excellent intra-rater reliability in using this software to assess the CSA of the LMM^8^. The assessor was blinded to any patient details and their pathology coding outcomes prior to and while acquiring the muscle measurements.

Measurements of the LMM commonly utilize total CSA and/or total muscle or fat CSA to assess atrophy or fatty degeneration, but proportionate muscle change appears to be a more promising method for finding true muscle morphology/quality alternations^10^. Focusing on the proportionate CSA of “pure” muscle, we implemented a repeatable approach to more objectively identify muscle cut-off values that also accounted for variations in image intensity between patient’s images and other inherent challenges in LMM analysis^8^.

### MRI muscle measurement method

**Figure 1:**
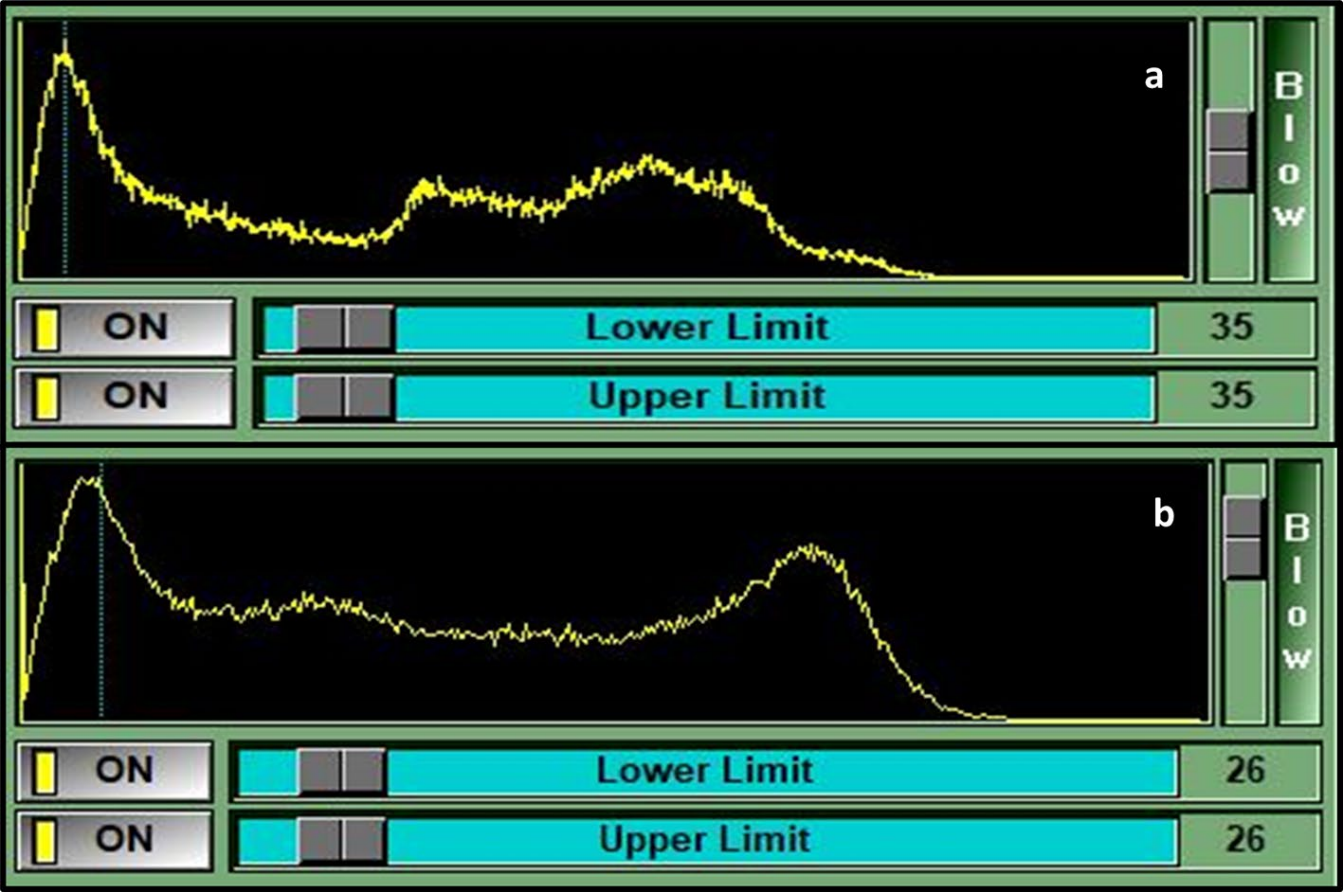
Muscle histograms. Example of two histograms demonstrating single (**a**) and triple (**b**) muscle peak values. Vertical dotted lines intersect the histogram to indicate the peak muscle signal values (i.e., 35 & 26).

For this analysis, the muscle cut-off value was defined as the maximum muscle signal intensity peak within the image histogram (Fig. 1a). This method took into account all muscle visible on the image (including psoas, erector spinae, and multifidi muscles) to reduce the errors in measurement values which occur when significant, isolated atrophy of the LMM is present. Once that peak was identified, its value was set as the upper range for muscle signal, with the signal values to the right of the peak encompassing any fat and remaining tissues. Importantly, this peak value was used for determining a reproducible cut-off point of the “purest” muscle for measurement purposes across all imaging cases, not to precisely define pure versus degraded muscle.

While the above noted method was used for most images, occasionally tightly packed double or triple peaks of similar height were present, in which case the peak with the highest signal (i.e., furthest to the right) was used as the cut-off value (Fig. 1b). In approximately 5% of cases, there was very little pure muscle anywhere on the image and thus no obvious muscle peak. In these cases, the muscle cut-off value was visually estimated and cross-checked for appropriateness during the CSA analysis.

**Figure 2:**
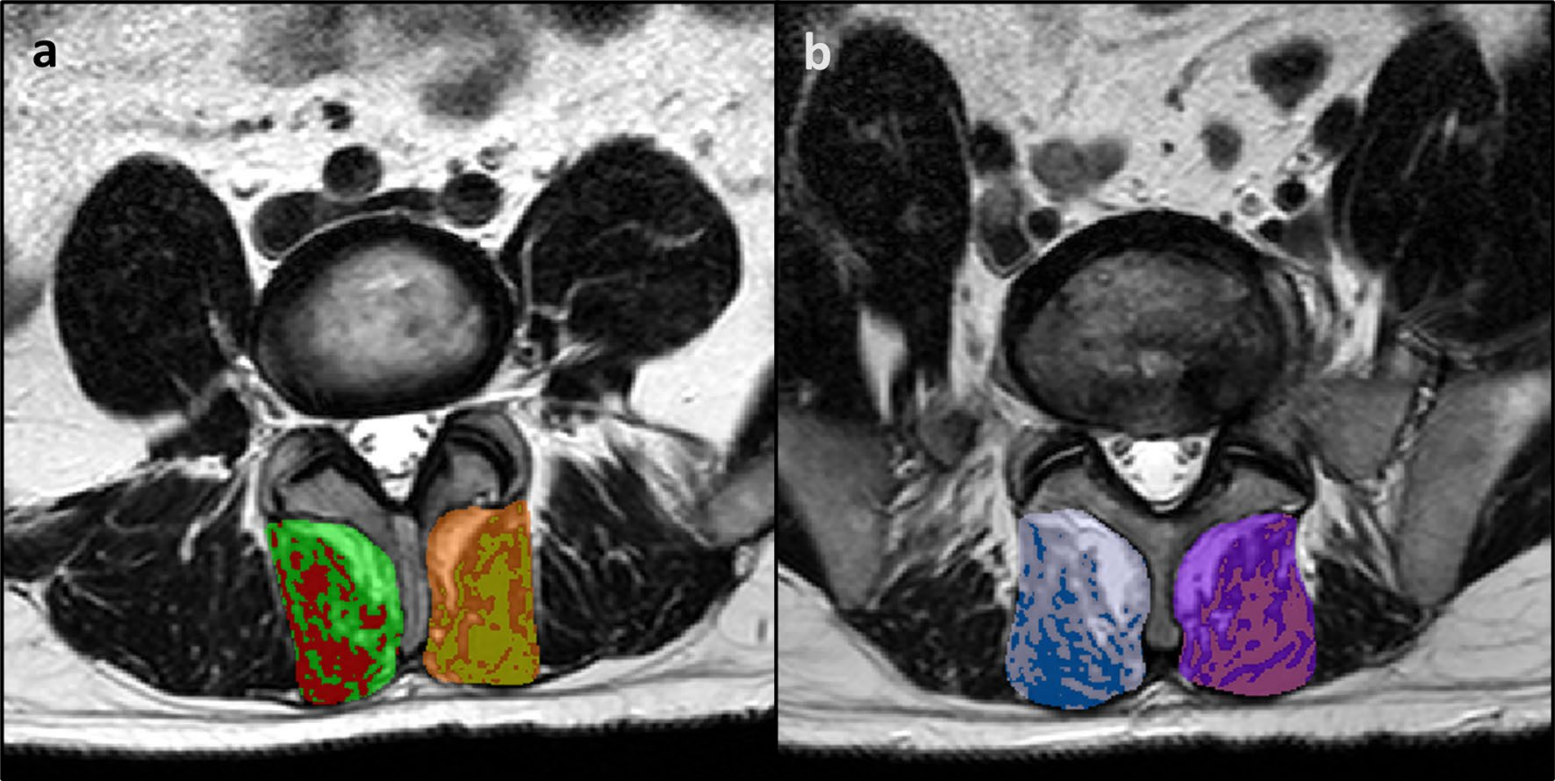
Multifidus muscle total CSA and % MCSA. (**a**) bilateral total CSA (L4), with red and gold highlights indicating the right and left peak muscle CSA, respectively; (**b**) bilateral total CSA (L5), with blue and pink highlighting the right and left peak muscle CSA, respectively. *Note* This method did not select all *potential* muscle signal; only the “purest” muscle tissue was highlighted.

Once the peak muscle value was determined, the LMM total CSAs were outlined using protocols developed previously^8^, with each LMM outlined by following the posterolateral margins of the spinous process and lamina to the facet joint region, descending along the cleavage plane separating the multifidus and longissimus muscles, then medially along the posterior fascial margin to the spinous process (Fig. 2a,b). If any LMM margins were not clear on the selected image, adjacent images from the original imaging series were reviewed to help localize the correct margins. Peak muscle versus remaining tissue CSAs were then determined (Fig. 2a,b), and the proportion (from 0.0 to 1.0) of peak muscle CSA was calculated [peak muscle CSA (cm)/total CSA (cm) = proportion muscle CSA [reported as percentage CSA (% MCSA)]]. The average L4 *and* L5% MCSA values and the worst % MCSA values for L4 *or* L5 were determined, with right or left-sided values applied to right or left-sided pathology analysis (i.e., right vs left-sided facet arthrosis or disc herniation apex) and bilateral values used for the remaining central and combined pathology analyses.

To assess intra-rater reliability for the muscle measurement method, cases were randomly selected for each spinal level (100 at L4; 100 at L5) from the initial patient pool using a computer-generated random numbers table. The muscle side to be measured from each selected image was randomly chosen, resulting in 52 right-sided and 48 left-sided muscle assessments per spinal level. Re-evaluation followed a minimum three-month waiting period, and the rater was blinded to all previous measures during the reliability analysis.

### Statistical analysis

We described continuous data (muscle outcomes) using means and standard deviations, and categorical data (degenerative MRI-findings) using frequency distributions (counts and percentages). Intra-rater reliability for % MCSA measures was assessed using intraclass correlation coefficients (ICC), with a two-way random effects, absolute agreement, single-measures model. ICCs with 95% confidence intervals and *p* values were reported, with intra-rater ICC values greater than 0.90 considered excellent^30^. To explore the cross-sectional associations between lumbar degenerative pathologies and the average and worst % MCSA outcomes at L4 and L5, we conducted univariable and multivariable linear regression models, adjusting for covariates age, sex and BMI, as they have been shown to have a significant association with altered paraspinal muscle size or fatty replacement and with degenerative conditions^13,24,31–35^. Model results were reported using unstandardized beta coefficients and 95% confidence intervals.

All hypotheses were two-sided and significance levels for all analyses was set at α = 0.05. Regression models were analyzed using Stata I/C version 17.0 (StataCorp, College Station, TX) and ICCs calculated with SPSS version 24 (IBM, IL, USA).

## Results

**Figure 3:**
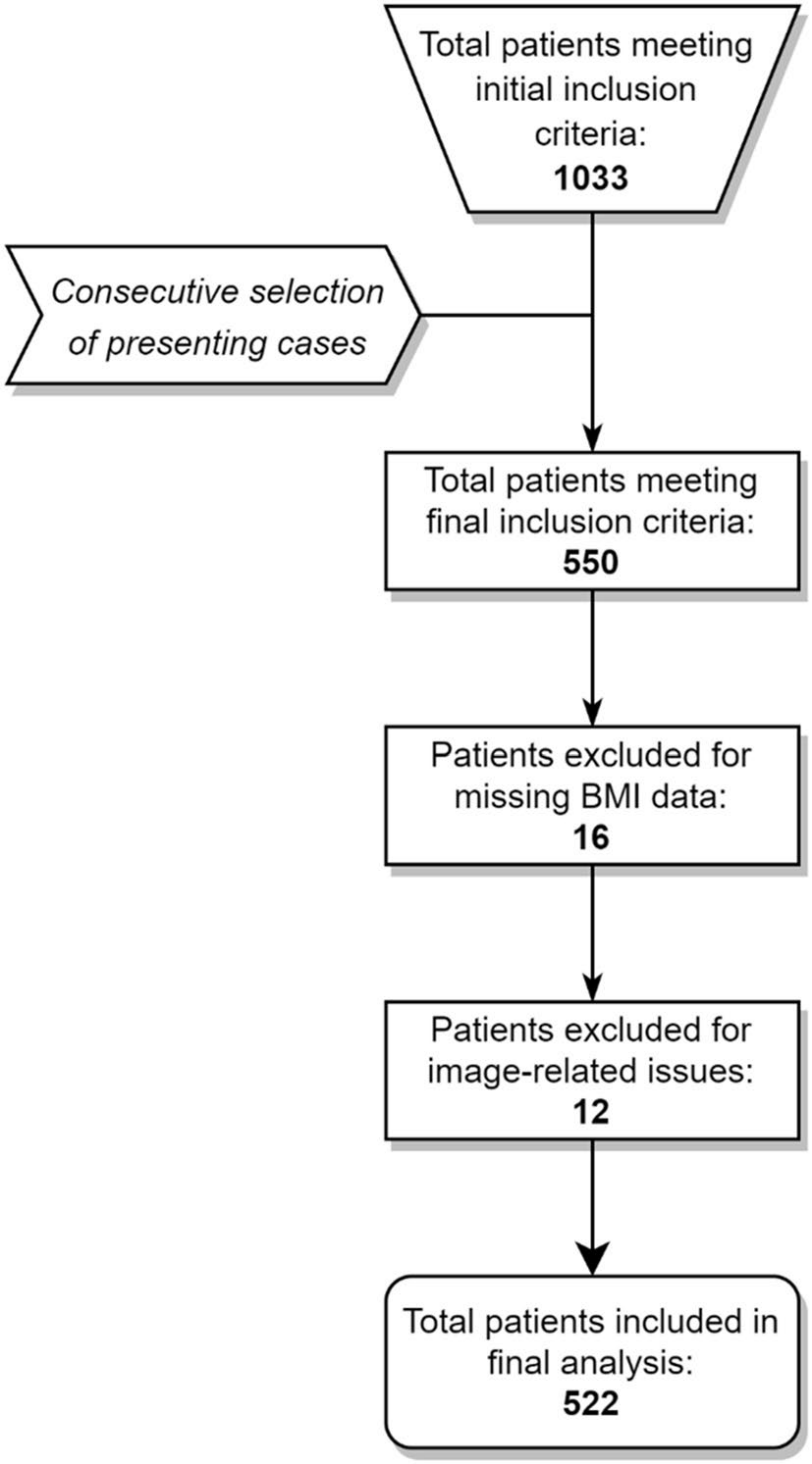
Pathology case selection flowchart.

A total of 522 cases met the criteria for inclusion in the final analysis (Fig. 3), equating to 2088 individual LMM measurements. Descriptive and summary data are provided in Tables 2 and 3. The average % MCSA mean was 35.1% across locations and ranged from 3.3% up to 72.6%. The worst % MCSA mean across locations was 30.5%, with a range of 2.3–69.3%. For both criteria, the left-sided muscles measured ~ 1.3% smaller than the right. Across all outcomes, the average and worst % MCSA reduced in the presence of each type of pathology, and generally reduced as the number of pathologies present increased.

**Table 2:** Descriptive statistics.

	Mean (SD)	Min–Max
**Covariates (n = 522)**		
Age (at 1st visit (years))	43.62 (9.8)	17–72
Age: Female (n = 294)	43.70 (10.1)	18–72
Age: Male (n = 228)	43.51 (9.5)	17–63
BMI (kg/m^2^)	26.51 (4.7)	17.5–42.7
BMI: Female (n = 294)	26.44 (5.3)	17.5–42.7
BMI: Male (n = 228)	26.59 (3.7)	18.4–39.7
**Muscle variables (n = 522)**		
Average % MCSA (L4 & L5; right)	35.69 (12.5)	5.04–72.55
Average % MCSA (L4 & L5; left)	34.48 (12.2)	3.33–69.28
Average % MCSA (L4 & L5; bilateral)	35.09 (11.9)	4.63–67.56
Worst % MCSA (L4 or L5; right)	32.08 (12.4)	2.26–69.28
Worst % MCSA (L4 or L5; left)	30.67 (11.9)	2.68–67.45
Worst % MCSA (L4 or L5; bilateral)	28.62 (11.8)	2.26–60.8

% MCSA, proportion of peak muscle cross-sectional area; SD, standard deviation.

**Table 3:** Descriptive summary of average and worst % MCSA (L4, L5) stratified by spinal pathology categories.

Category	N (%)	Mean (SD)	Mean (SD)
	(Total: 522)	Average % MCSA	Worst % MCSA
**Disc degeneration: total levels > grade1**			
None	272 (52.1)	37.1 (11.4)	30.6 (11.4)
1 level	151 (28.9)	34.1 (11.7)	27.7 (11.9)
2 + levels	99 (19.0)	31.1 (12.5)	24.7 (11.7)
**High intensity zone: total levels affected**			
None	232 (44.4)	36.4 (12.2)	30.1 (12.2)
1 level	193 (37.0)	34.6 (11.3)	27.8 (11.3)
2 + levels	97 (18.6)	32.9 (12.1)	26.7 (11.4)
**Disc bulge: present any level**			
Not present	413 (79.1)	35.9 (12.1)	29.3 (12)
Present	109 (20.9)	31.9 (10.7)	25.8 (10.5)
**Disc herniation: worst changes any location**			
None to mild	432 (82.8)	35.6 (11.9)	29 (11.8)
Moderate to severe	90 (17.2)	32.4 (12)	28.5 (11.9)
**Modic type 1: worst changes any level**			
None to mild	409 (78.4)	35.9 (12)	29.5 (12)
Moderate to severe	113 (21.6)	32 (11.3)	25.5 (10.5)
**Modic type 2: worst changes any level**			
None to mild	418 (80.1)	36.4 (11.8)	30 (11.8)
Moderate to severe	104 (19.9)	29.8 (10.8)	23.1 (10.3)
**Endplate defects: present any level**			
Not present	388 (74.3)	35.7 (12.1)	29.1 (12)
Present	134 (25.7)	33.2 (11.2)	27.1 (11)
**Osteophytes: total levels affected**			
None	244 (46.7)	37 (11.6)	30.4 (11.8)
1 level	146 (28.0)	33.6 (11.8)	26.8 (11.4)
2 + levels	132 (25.3)	33.3 (12.3)	27.3 (11.9)
**Facet arthrosis (right): total levels > grade1**			
None	263 (50.4)	37.9 (12.4)	34.4 (12.4)
1 level	142 (27.2)	34.4 (11.9)	30.6 (11.8)
2 + levels	117 (22.4)	32.2 (12.4)	28.7 (12.2)
**Facet arthrosis (left): total levels > grade1**			
None	270 (51.7)	36.7 (11.6)	32.8 (11.4)
1 level	148 (28.4)	32.1 (12.2)	28.1 (11.8)
2 + levels	104 (19.9)	32.1 (12.7)	28.9 (12.5)
**Combined pathologies**			
< 2	122 (23.4)	39.4 (11.7)	33 (11.8)
2	93 (17.8)	37.2 (11.1)	30.1 (11.3)
3	94 (18.0)	36.4 (12.4)	29.6 (12.7)
4	75 (14.4)	31.3 (10.9)	25.1 (10.5)
5	77 (14.6)	32.7 (11.2)	26.6 (10.8)
> 5	61 (11.7)	28.9 (10.4)	22.9 (9.9)

% MCSA, proportion of peak muscle cross-sectional area; SD, standard deviation.

For intra-rater reliability, 100 cases were randomly selected for each spinal level (L4 and L5), with a relatively even distribution of both right and left-sided muscles per level (52 right; 48 left). Reliability (ICC (95%CI)) for % MCSA was similar by spinal level and side, and excellent (> 0.9) across all measures [L4 (right) = 0.945 (0.887–0.971), (left) = 0.948 (0.899–0.972); L5 (right) = 0.953 (0.914–0.974), (left) = 0.942 (0.844–0.974)]. All results were significant at *p* =< 0.001.

Univariable analysis of individual pathologies (Table 4) identified a significantly lower average and worst % MCSA criteria for all variables except single level HIZs (average %), and disc herniations and endplate defects (worst %). The greatest differences were associated with Modic type 2 marrow changes under both muscle criteria (− 6.6, − 6.8), followed by DD (− 6.1, − 5.9) and right-sided facet arthrosis (− 5.7, − 5.8). When levels of involvement were assessed, the trend was towards further lowering of % MCSA when more levels of involvement were present.

**Table 4:** Linear regression [individual pathologies]: average and worst % MCSA.

Pathology variable	Univariable models		Adjusted models^1^	
	β	95% CI	β	95% CI
***Average %—L4 & L5***				
**Disc degeneration**				
1 level	− 3.06	− 5.39, − 0.72^†^	− 1.63	− 3.48, 0.21
2 + levels	− 6.06	− 8.76, − 3.36^†^	− 4.51	− 6.72, − 2.3^†^
**High intensity zones**				
1 level	− 1.86	− 4.13, 0.41	− 0.38	− 2.19, 1.43
2 + levels	− 3.57	− 6.39, − 0.75^†^	0.04	− 2.25, 2.32
Disc bulge: any level	− 3.97	− 6.47, − 1.47^†^	− 1.62	− 3.62, 0.39
Disc herniation: moderate to severe	− 3.20	− 5.92, − 0.48^†^	− 3.66	− 5.81, − 1.52^†^
Modic type 1: moderate to severe	− 3.91	− 6.38, − 1.44^†^	− 1.64	− 3.62, 0.33
Modic type 2: moderate to severe	− 6.58	− 9.08, − 4.08^†^	− 4.06	− 6.09, − 2.04^†^
Endplate defects: any level	− 2.54	− 4.88, − 0.21^†^	− 2.74	− 4.58, − 0.91^†^
**Osteophytes**				
1 level	− 3.35	− 5.78, − 0.93^†^	− 0.22	− 2.26, 1.81
2 + levels	− 3.68	− 6.18, − 1.17^†^	− 0.37	− 2.63, 1.89
**Facet arthrosis (right)**				
1 level	− 3.50	− 6.01, − 0.99^†^	− 1.86	− 3.93, 0.22
2 + levels	− 5.77	8.44, − 3.09^†^	− 4.02	− 6.26, − 1.78^†^
**Facet arthrosis (left)**				
1 level	− 4.66	− 7.06, − 2.25^†^	− 3.06	− 4.99, − 1.13^†^
2 + levels	− 4.57	− 7.29, − 1.86^†^	− 3.02	− 5.23, − 0.82^†^
***Worst %—L4 or L5***				
**Disc degeneration**				
1 level	− 2.87	− 5.18, − 0.56^†^	− 1.45	− 3.28, 0.39
2 + levels	− 5.90	− 8.57, − 3.23^†^	− 4.32	− 6.52, − 2.12^†^
**High intensity zones**				
1 level	− 2.37	− 4.62, − 0.13^†^	− 0.89	− 2.69, 0.90
2 + levels	− 3.43	− 6.21, − 0.64^†^	0.16	− 2.11, 2.42
Disc bulge: any level	− 3.50	− 5.98, − 1.03^†^	− 1.14	− 3.13, 0.86
Disc herniation: moderate to severe	− 0.55	− 3.24, 2.14	− 0.96	− 3.09, 1.17
Modic type 1: moderate to severe	− 3.99	− 6.43, − 1.55^†^	− 1.74	− 3.70, 0.22
Modic type 2: moderate to severe	− 6.85	− 9.32, − 4.38^†^	− 4.35	− 6.36, − 2.35^†^
Endplate defects: any level	− 2.01	− 4.33, 0.30	− 2.18	− 4.00, − 0.36^†^
**Osteophytes**				
1 level	− 3.60	− 6.00, − 1.20^†^	− 0.40	− 2.42, 1.62
2 + levels	− 3.09	− 5.57, − 0.61^†^	0.37	− 1.87, 2.62
**Facet arthrosis (right)**				
1 level	− 3.74	− 6.24, − 1.24^†^	− 1.99	− 4.06, 0.08
2 + levels	− 5.68	− 8.34, − 3.01^†^	− 3.78	− 6.01, − 1.54^†^
**Facet arthrosis (left)**				
1 level	− 4.69	− 7.04, − 2.34^†^	− 3.16	− 5.05, − 1.28^†^
2 + levels	− 3.84	− 6.49, − 1.18^†^	− 2.38	− 4.53, − 0.23^†^

^1^Adjusted for age, sex, and BMI. ^†^Significant difference from reference (for each pathology variable, the reference category is either the absence of the condition, or the absence of moderate to severe findings for that condition). β, unstandardized beta coefficient; CI, confidence interval; % MCSA, proportion of peak muscle cross-sectional area.

The adjusted linear regression models (Table 4) showed that DD (two or more levels), Modic type 2 marrow changes, any endplate defects, and facet arthrosis (two or more levels on the right; any level on the left) remained associated with lower average and worst % MCSA, after controlling for age, sex, and BMI. The presence of moderate to severe disc herniations was associated with a significantly lower average % MCSA only. Disc degeneration, Modic type 2 marrow change, and right-sided facet arthrosis had the strongest association with % MCSA, with differences ranging from − 4.0 to − 4.5, while endplate defects demonstrated the lowest significant association at − 2.2. Right-sided % MCSA was lowest with multi-level, right-sided facet arthrosis, while left-sided % MCSA was lowest with single level, left-sided facet arthrosis.

**Figure 4:**
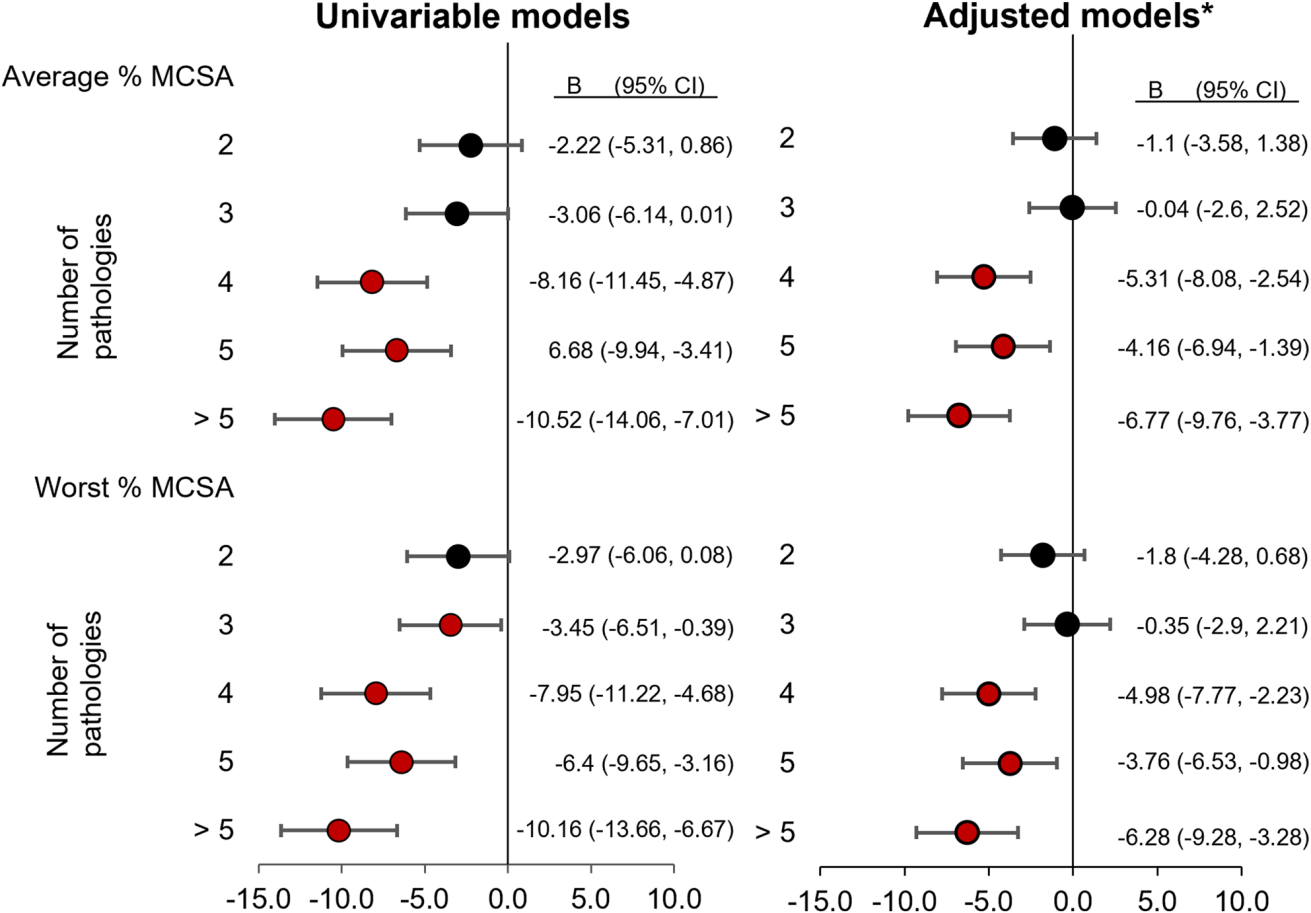
Linear regression [combined pathologies]. *Adjusted for age, sex, and BMI. % MCSA = proportion of peak muscle cross-sectional area; B = unstandardized beta coefficient; CI = confidence interval. NB: for each model, the reference category is < 2 pathologies present.

Figure 4 displays the combined pathology % MCSA outcomes for the univariable and adjusted linear regression models. The unadjusted model showed significantly lower average and worst % MCSA measures once 4 and 3 different pathologies were present, respectively. An overall trend towards smaller % MCSA was noted as the number of different pathologies increased, with differences below − 10.0 associated with the presence of 6 or more pathologies. After adjusting for age, sex, and BMI, only the presence of 4 or more pathologies was associated with significantly lower average and worst % MCSA. Again, the greatest difference was noted with the presence of 6 or more pathologies, being below − 6.0 for both criteria. The greatest differences in both adjusted combined pathology models were about − 2.0 more than that seen with any individual pathologies.

## Discussion

Based on our search of the literature, we believe this study is the first large scale evaluation for potential associations between altered LMM morphology and a diverse collection of common degeneration-related lumbar spine conditions, both individually and in aggregate. All unadjusted estimates demonstrated a statistically significant reduction in the average and/or worst % MCSA in the presence of pathology, typically worsening as their severity or distribution increased. Except for the presence of HIZs, this held true for the adjusted analyses as well.

When specifically considering those conditions with a potentially direct neuromechanical relationship with the LMM (i.e., herniations with direct neural compression, facet arthrosis, vertebral osteophytes) there were mixed results, with osteophytes showing no significant association, disc herniations demonstrating a mixed outcome (significant for the average, but not for the worst % MCSA), and facet arthrosis consistently showing significance when two or more levels were affected. While some studies have shown an association between lumbar disc herniations and paraspinal muscle atrophy^20,31^, the absence of a consistently significantly smaller LMM % MCSA in patients with disc herniations would be in line with recent systematic reviews which identified no consistent associations in this regard^10,25^. There has been no consensus regarding any associations between facet arthrosis and altered muscle quality, although most studies have used computed tomography for assessing this condition^10,32,36,37^.

Conversely, three conditions that did demonstrate a significant association with smaller % MCSA have no clear neuromechanical mechanism: multi-level, moderate to severe DD, moderate to severe Modic type 2 changes at any level, and endplate defects at any level. Associations noted between LMM morphology changes and DD, Modic changes, and endplate defects are consistent with the findings of several studies (particularly when focusing on muscle quality as opposed to overall volume)^13,14,18,19,24,35,38,39^, although a few studies have noted no consistent associations between these conditions and altered paraspinal muscles^20,32,40,41^. While we did find an initial association with LMM degradation and Modic type 1marrow changes consistent with Atci, et al.’s 2020 study^13^, this association failed to remain significant after adjusting for covariates.

The clinical relevance of any individual pathology associations with muscle quality is unknown, and it is possible any noted associations were coincidental. Nevertheless, there are potential explanations for an underlying complex inter-relationship between the LMMs and the anatomical/pathological findings surrounding them. For example, a consistent association between disc degeneration, Modic type 1 or 2 marrow changes, and vertebral endplate defects has been identified by several authors^18,38,39,42^, leading Teichtahl et al.^39^, to suggest that segmental spinal degeneration could be considered as a “whole organ” disease. If so, then any significant associations between alterations of the disc, bone marrow, cartilage endplates, and supporting paraspinal muscles at individual or adjacent spinal levels would make sense pathophysiologically. Alternatively, over time degenerative processes (such as DD / Modic changes) may potentially contribute to recurring episodes or long periods of back pain, which could inhibit muscle activity and eventually impact on LMM quality^43,44^. However, any potential casual explanations are still speculative, and while studies suggesting a causal pathway from spinal pathology to muscle degeneration do exist in animal models, a causal direction in humans is still unclear^45^. Since a consistent sequence for the appearance of these various changes has yet to be identified, no causal direction between the individual degenerative pathologies investigated and changes to muscle morphology can be implied from our findings.

Similar to Hancock et al.’s study demonstrating a positive association between the number of MR findings and the risk of LBP outcomes^29^, in our study, as the number of different pathologies per patient increased, the peak muscle percentage became progressively smaller. This became significant once 4 pathologies were present (adjusted models), supporting a potential dose–response relationship between spinal pathology and LMM morphology. Although we did not look at the specific combinations of pathologies contributing to the aggregate effect, it is possible that individual conditions that were significantly or insignificantly associated with altered LMM changes could be contributing to any aggregate pathology associations. Even so, the greatest differences in % MCSA for both adjusted, combined pathology models was between − 6.3 and − 6.8, ~ 2.0 greater than any individual pathologies. This raised the question as to why some pathologies were associated with ~ 4.0 smaller % MCSA individually, while a similar degree of difference didn’t occur in the combined models until 4 pathologies were reached. The two most probable explanations are (1) although each pathology variable was assessed individually, it is possible that more than one type of pathology was present in the background, and (2) the individual pathology analysis took into account the number of levels affected, whereas the combined pathologies analysis was based on any one level being affected. These explanations suggest it would be appropriate to consider the severity, type, and total levels affected by pathology, as well as the total number of different pathologies present, when evaluating the degree of muscle degradation associated with spinal pathology. Multiple pathology outcomes were associated with significantly lower pure muscle proportion. However, the clinical importance of the amount of difference seen cannot be determined from our study and as such no clinical implications are inferred.

Some limitations with this study were identified and addressed where possible to reduce their impact on the results. While the revised method used to determine muscle signal cut-off values provided a reliable and objective approach, there were still a small percentage of cases that required a visual estimation of the muscle cut-off value. This interjected some subjectivity into the process; however, as exclusion of these cases would have removed valuable information regarding muscle/pathology associations at the more severe end of the muscle degradation spectrum, to retain these cases was deemed more appropriate. As the study participants comprised patients with current low back and/or lumbar spine-related leg pain, these results may not generalize to asymptomatic populations with spinal degeneration. The clinical data collection process did not allow for specific identification of patients with an underlying systemic neurological or myopathic disorder; however, our focus on patients presenting with a primary low back-related complaint should have effectively excluded these systemic disorders. There was a potential for unmeasured confounding from activity-related factors, such as physical activity and physically demanding nature of work, although the impact of these factors on our final outcomes may have been partially mitigated through opposing effects on spinal degeneration and muscle quality. Finally, as this was a cross-sectional study, we were unable to capture the full complexity of the relations between spinal pathology and altered muscle morphology and any changes that may occur over time. Therefore, high-quality longitudinal data is needed to advance this line of research.

## Conclusion

This study provided a cross-sectional analysis of associations between degenerative spinal pathologies and LMM morphology. The LMM measurement method applied showed excellent intra-rater reliability. Our hypothesis that associations will be demonstrated between lumbar region degenerative pathology and lower proportions of pure multifidus muscle was supported. Adjusting for age, sex, and BMI, several pathologies were associated with smaller proportions of muscle, particularly multi-level DD, multi-level facet joint arthrosis, and moderate to severe Modic type 2 marrow changes. These associations remained as the number of different pathologies reached 4 and greater, supporting a potential dose–response relationship between spinal pathology and LMM morphology. Future longitudinal studies could help determine if these associations between spinal and muscle findings are both part of the same degenerative process, result from prior injury or other common antecedent events, or due to an actual directional relationship. For future assessments of associations between LMM degradation and degenerative conditions in the lumbar spine, the type, severity, total levels affected, and the total number of different pathologies present should all be considered.

## Data Availability

The imaging and clinical datasets analyzed during the current study are not publicly available as they are patient files covered by EU privacy legislation, requiring permission from the database manager to access. Upon reasonable request to the corresponding author, deidentified datasets generated from this study may be made available with database manager approval.

## Abbreviations

CSA: Cross-sectional area
HIZ: High intensity zone
LMM: Lumbar multifidus muscle
DD: Disc degeneration
% MCSA: Proportion of peak muscle cross-sectional area
